# A comparative physical map reveals the pattern of chromosomal evolution between the turkey (*Meleagris gallopavo*) and chicken (*Gallus gallus*) genomes

**DOI:** 10.1186/1471-2164-12-447

**Published:** 2011-09-09

**Authors:** Yang Zhang, Xiaojun Zhang, Thomas H O'Hare, William S Payne, Jennifer J Dong, Chantel F Scheuring, Meiping Zhang, James J Huang, Mi-Kyung Lee, Mary E Delany, Hong-Bin Zhang, Jerry B Dodgson

**Affiliations:** 1 Department of Soil and Crop Sciences, Texas A&M University, College Station, TX 77843, USA; 2 Department of Animal Science, University of California, Davis, CA 95616, USA; 3 Department of Microbiology & Molecular Genetics, Michigan State University, East Lansing, MI 48824, USA

## Abstract

**Background:**

A robust bacterial artificial chromosome (BAC)-based physical map is essential for many aspects of genomics research, including an understanding of chromosome evolution, high-resolution genome mapping, marker-assisted breeding, positional cloning of genes, and quantitative trait analysis. To facilitate turkey genetics research and better understand avian genome evolution, a BAC-based integrated physical, genetic, and comparative map was developed for this important agricultural species.

**Results:**

The turkey genome physical map was constructed based on 74,013 BAC fingerprints (11.9 × coverage) from two independent libraries, and it was integrated with the turkey genetic map and chicken genome sequence using over 41,400 BAC assignments identified by 3,499 overgo hybridization probes along with > 43,000 BAC end sequences. The physical-comparative map consists of 74 BAC contigs, with an average contig size of 13.6 Mb. All but four of the turkey chromosomes were spanned on this map by three or fewer contigs, with 14 chromosomes spanned by a single contig and nine chromosomes spanned by two contigs. This map predicts 20 to 27 major rearrangements distinguishing turkey and chicken chromosomes, despite up to 40 million years of separate evolution between the two species. These data elucidate the chromosomal evolutionary pattern within the *Phasianidae *that led to the modern turkey and chicken karyotypes. The predominant rearrangement mode involves intra-chromosomal inversions, and there is a clear bias for these to result in centromere locations at or near telomeres in turkey chromosomes, in comparison to interstitial centromeres in the orthologous chicken chromosomes.

**Conclusion:**

The BAC-based turkey-chicken comparative map provides novel insights into the evolution of avian genomes, a framework for assembly of turkey whole genome shotgun sequencing data, and tools for enhanced genetic improvement of these important agricultural and model species.

## Background

Turkey, *Meleagris gallopavo *(MGA), is second to chicken (*Gallus gallus*, GGA) as an agriculturally important avian species in the U.S. and globally [1]. Phylogenetic analyses suggest that the last common ancestor to the turkey and chicken lived between 20 and 40 million years ago [2,3]. Genetic analysis and the requisite tools for modern turkey breeding have hitherto focused on developing a genetic linkage map with limited physical information. Turkey genome research has lagged behind and, to some extent, depended upon our understanding of the chicken genome. Karyotype analysis demonstrated that the chromosomes of turkey are substantially similar to those of chicken [reviewed in 4]. Turkey has a diploid chromosome number of 80, as opposed to 78 for chicken. Most chicken chromosomes appear to correspond to single orthologous turkey chromosomes, except for GGA2, orthologous to MGA3 and MGA6, and GGA4, orthologous to MGA4 and MGA9, probably due to centric fission events in the turkey lineage [4]. The availability of the complete chicken genome sequence and its associated resources [5] provided the opportunity to analyze the turkey genome and its evolutionary relatedness to that of the chicken in much greater depth.

An important step towards the comprehensive analysis of a large genome is the generation of high-quality, well-anchored physical maps [6-9]. Such maps have been widely used to effectively integrate genomic tools for high-resolution genome mapping, marker-assisted breeding, positional cloning of genes, and quantitative trait locus (QTL) detection [10,11]. Simultaneously, physical maps provide desirable platforms for whole genome sequencing and assembly [12-15] and large-scale comparative genomics. Various strategies for creating such maps have been employed, but the use of multiple independent data sources is desirable for cross-checking alignments and minimizing errors. Bacterial artificial chromosome (BAC) fingerprints and BAC-end sequences (BES) together with genetic maps and cytogenetic analysis provide an efficient strategy for building robust whole-genome physical maps for large genomes. For example, Gregory et al. [8] produced a detailed comparative physical map of the mouse and human genomes by combining BAC-end sequencing with a whole-genome BAC contig map created using BAC fingerprints, revealing a high level of local colinearity between these two genomes. Fujiyama et al. [16] constructed a clone-based comparative map of the human and chimpanzee genomes using paired chimpanzee BES aligned with the human genome sequence. Larkin et al. [17] built a cattle-human comparative map using cattle BES and the human genome sequence. Roberto et al. [18] reported a refined gibbon genome comparative map with respect to the human genome by combining BES and fluorescence in situ hybridization (FISH) analysis. Wei et al. [19] generated a sequence-ready physical map of maize and aligned it to the genome of rice, revealing its complex evolutionary history. Gu et al. [20] constructed a BAC-based physical map of *Brachypodium distachyon *and compared it with the rice and wheat genomes, providing an important resource for the completion of the *Brachypodium *genome sequence and grass comparative genomics.

Turkey has a genome size similar to that of the chicken at 1,100 million base pairs (Mb) per haploid (1C) genome [5]. To develop tools essential for continued genetic improvement of this species, DNA marker-based genetic maps have been developed and aligned with those of the chicken [21-26]. Recently, multi-platform next-generation whole genome shotgun sequencing of the domestic turkey has been carried out [13], and that sequence was assembled, in part, using a preliminary version of the map that we describe below. However, further advances, such as the development of DNA markers for fine mapping in a region of interest, isolation of clones containing a gene and/or QTL by positional cloning, finished quality whole genome sequence assembly, and large-scale comparative genomics analyses with other *Galliformes *genomes, require the powerful infrastructure derived from a detailed physical comparative BAC map.

Here, we report a genome-wide BAC-based physical and comparative map of the turkey genome, integrated using an average of one sequence-tagged site per 25 kilobase pairs (kb). Alignment of the turkey physical map with the chicken genome sequence identified 20 to 27 major chromosome rearrangement events that occurred during the separate evolution of the turkey and chicken genomes, most of which were shown to be inversions. These results suggest that genomes within the *Phasianidae *have remained remarkably stable throughout their history and reveal interesting trends in the evolution of avian chromosomes.

## Results and discussion

### Turkey BAC contig physical map

A total of 85,208 clones were fingerprinted for physical map assembly, randomly selected from two large-insert turkey BAC libraries, CHORI-260 and TKNMI (see Methods), the latter of which was generated in this study. These clones together cover the turkey haploid genome by about 13.7-fold, with 8.1- and 5.6-fold coverage from CHORI-260 and TKNMI, respectively (Table 1). Furthermore, we sequenced 43,238 BAC ends randomly selected from the two BAC libraries and hybridized the libraries with over 3,500 overgo probes designed from turkey BES, microsatellite markers and genes, chicken genes, and other regions of the chicken genome demonstrating high evolutionary conservation to facilitate map construction and comparative genomics.

**Table 1:** Summary of the turkey BAC libraries used in this study

Library name	CHORI-260	78TKNMI
Cloning vector	pTARBAC2.1	pECBAC1

DNA source	Whole blood	Whole blood

Restriction enzyme used	*Eco*RI	*Mbo*I

Average insert size	190 kb	160 kb

Total 384-well plates	192 (73,728 clones)	120 (46,080 clones)

Genome coverage	12.7 x	6.7 x

Clones fingerprinted	46,808	38,400

Genome coverage of clones fingerprinted	8.1 x	5.6 x

We assembled the turkey physical map from BAC fingerprints, BES and hybridization results independently using two approaches. In the first approach, contigs were assembled automatically using FingerPrinted Contigs (FPC) version 9.3 [27] and then edited, verified and extended as follows. Only those BACs with fingerprints of 16 or more bands were selected for contig construction. As a result, a total of 74,013 BACs, covering the turkey haploid genome 11.9-fold, were validated for map assembly. Every FPC contig was manually checked for potential chimeric contigs based on BAC fingerprint patterns. All questionable contigs were split and re-assembled at a higher stringency using cutoff values ranging from 1e-11 to 1e-09 (FPC DQer function). Then, to identify potential junctions between contigs, the entire fingerprint database was searched for matches to terminal clone fingerprints of every contig using the end to end function of FPC with cutoff values ranging from 1e-20 to 1e-07. Contigs were merged only if terminal clones shared 10 or more bands and their overall fingerprint patterns supported the merge. Next, 2,551 DNA markers assigned to 15,683 BACs by overgo hybridization (Methods) were incorporated into the physical map contigs. Contigs were merged using cutoff values between 1e-08 and 1e-04 if they shared markers and their terminal overlapping band patterns supported the merge. Then we incorporated 28,385 BES including 11,829 mate-pairs into the physical map. Finally, without any further merges, singletons were added if there were overlaps with one or more clones in a contig using cutoff values between 1e-20 and 1e-05.

The map assembly strategies described above reduced the total number of contigs in the turkey physical map to 720 from 8,870, containing a total of 55,192 clones, whereas the remaining 18,821 clones remained as singletons. Each contig contains 2 to 955 clones with an average of 77 clones per contig. The contigs span from 78 to 26,130 kb, with an average physical length of 2,317 kb (Table 2). The 720 contigs consisted of 575,144 consensus bands, estimated to span approximately 1,668 Mb. See Additional file 1: Table S1 for a list of the contigs of the turkey physical map. Table 2 summarizes characteristics of the resultant turkey BAC contig map. We aligned the physical map contigs to the chicken genome sequence (Methods). Of the 720 contigs, 516 (71.7%) spanning 1,609 Mb or 96.4% of the total physical length of the turkey physical map could be aligned to the chicken genome sequence, based on the hybridization of 2,551 unique probes to 15,683 BACs and the BLASTN alignment of 28,385 turkey BES as anchors.

**Table 2:** Summary of the turkey genome physical map and its integration with the chicken genome

Clones used in the physical map construction	74,013 (11.9 x)
Contigs assembled	720

Singletons	18,821

Clones contained in the contigs	55,192 (8.9 x)

Questionable clones in the contigs	6,028 (8.1%)

Consensus bands of the contigs	575,144

Average band number per clone	53.9

Average clone number per contig	76.7

Physical length contribution per clone	30.3 kb

Average contig size	2,317 kb

Largest contig size	26.1 Mb (containing 995 clones)

BES contained in the physical map	28,385

Total physical length of the physical map	1,668 Mb

DNA markers/genes in the physical map	2,551

DNA markers/genes aligned to the chicken genome	2,551 (15,683 BAC assignments)

BES aligned to the chicken genome	28,385 (18,821 paired-ends)

Contigs assigned to the chicken chromosomes	516 (1,609 Mb)

The largest contig in the map consisted of 955 BAC clones, spanning more than 26 Mb in physical length. The average BAC contig size of the map is > 2 Mb with the N50 contig size being > 7.8 Mb. Coverage by BACs assembled into the map was 11.9-fold (Table 2). According to our previous studies [28-34], this coverage is expected to generate a high-quality physical map. Moreover, since the source clones for the map were selected from two BAC libraries constructed with different restriction enzymes (*Eco*RI and *Mbo*I), the resultant map is expected to have enhanced actual genome coverage [10]. The 720 contigs of the turkey physical map collectively span approximately 1,668 Mb in physical length, larger than the estimated 1,100-Mb turkey genome size by approximately 50%. The excess in total physical length is expected because FPC will not merge all truly overlapping contigs. These overlaps exist between contigs for which there are too few common restriction fragments to allow for a statistically significant merge, given the stringent criteria employed to minimize false positive overlaps.

### BES-based turkey-chicken BAC map

Based on the high frequency at which turkey BES were uniquely aligned to the chicken sequence assembly (Methods) and the availability of extensive data from overgo hybridization (with orthologous placements on the chicken sequence), we also generated a turkey BAC physical map using an alternative approach. Contigs were first assembled by BES alignment to the chicken genome and then merged using terminal overgo hybridization to a common BAC or common placement of terminal BACs in a single fingerprint contig. This approach provided a unique most likely orthologous alignment for 44,493 turkey BACs along the framework chicken sequence (Additional file 2: Table S2). Initial contigs were assembled from overlapping BAC clones, each of which was placed by two consistent alignments (a BES mate pair or one BES and one overgo hybridization), followed by merging the resultant contigs based on fingerprint contigs that were assembled independently of the BES data.

This second approach resulted in a comparative physical BAC map between turkey and chicken, consisting of only 74 turkey contigs (Table 3 and Additional file 3: Table S3). The comparative map spans 1,004 Mb of the chicken sequence, with an average contig size of 13.6 Mb, a N50 contig size of 30.9 Mb and a N90 contig size of 9.2 Mb. All but four of the mapped turkey chromosomes were spanned on this map by three or fewer contigs, with 14 chromosomes spanned by a single contig and nine chromosomes spanned by two contigs. Not surprisingly, the turkey Z chromosome is less contiguous, consisting of 11 contigs, due to the fact that females (ZW) were used for both the turkey BAC libraries and the chicken sequence assembly. The arrangement and number of contigs in our turkey Z map did not change when we used the more complete chicken Z sequence of Bellott et al. [35] rather than the WUGSC2.1/galGal3 assembly (Additional file 4: Table S4), although the sizes of contigs with internal repeats can change. Our MGA18, MGA27 and MGA30 maps also are likely to be incomplete due to partial and/or uncertain coverage of the orthologous chromosomes (GGA16, GGA25, and GGA28, respectively) in the chicken sequence assembly [36,37].

**Table 3:** Summary properties of comparative BAC contig map and rearrangements

MGA chromosome	Number of contigs	GGA orthologue	Total contig length (bp)	Average contig length (bp)	Number of major inversions
MGA1	9	GGA1	198,141,542	22,015,727	0

MGA2	1	GGA3	111,826,550	111,826,550	2

MGA3	1	GGA2q	100,502,741	100,502,741	1

MGA4	3	GGA4q	72,769,490	24,256,497	0

MGA5	2	GGA5	60,352,001	30,176,001	2

MGA6	1	GGA2p	51,981,596	51,981,596	0

MGA7	5	GGA7	36,281,918	7,256,384	1

MGA8	2	GGA6	34,867,379	17,433,690	4

MGA9	1	GGA4p	19,188,313	19,188,313	0

MGA10	2	GGA8	29,163,447	14,581,724	2

MGA11	2	GGA9	23,514,388	11,757,194	1

MGA12	1	GGA10	22,321,123	22,321,123	0-2*

MGA13	1	GGA11	21,290,332	21,290,332	1

MGA14	2	GGA12	19,602,960	9,801,480	1-4

MGA15	2	GGA13	17,856,723	8,928,362	2

MGA16	1	GGA14	15,894,242	15,894,242	1

MGA17	1	GGA15	12,928,248	12,928,248	0

MGA18	1	GGA16	68,068	68,068	ND

MGA19	1	GGA17	10,577,421	10,577,421	0

MGA20	1	GGA18	10,507,821	10,507,821	1

MGA21	1	GGA19	9,897,437	9,897,437	0

MGA22	3	GGA20	13,624,242	4,541,414	0

MGA23	1	GGA21	6,854,714	6,854,714	0

MGA24	3	GGA22	3,657,921	1,219,307	0

MGA25	2	GGA23	5,832,856	2,916,428	0-2*

MGA26	1	GGA24	6,430,646	6,430,646	0

MGA27	5	GGA25	2,143,571	428,714	ND

MGA28	2	GGA26	4,831,899	2,415,950	0

MGA29	3	GGA27	4,641,426	1,547,142	0

MGA30	2	GGA28	4,439,785	2,219,893	ND

MGAZ	11	GGAZ	72,157,792	6,559,799	1**

TOTALS	74		1,004,148,592	13,569,576	20-27

ND indicates that the comparative map and/or chicken sequence assembly are incomplete

*Pattern can be explained by two sequential inversions or centromere replacement

**There may be additional small rearrangements

### Comparison of mapping approaches

The two BAC contig map building approaches described above employed identical or overlapping data sets but differed in the order in which those data were used. The first approach built the initial physical map using FPC with BAC fingerprints only, followed by incorporating DNA overgo probe results (including linkage map markers) and BES alignments, whereas the second approach began by aligning turkey BACs into contigs along the chicken genome sequence based on consistent BES mate pair alignment or BES plus overgo hybridization alignment, followed by contig merging using fingerprint contig data and further hybridization or FISH analysis. This approach took advantage of the expected high level of homology between the chicken and turkey genomes, as confirmed by the very high rate of matching turkey BES to unique orthologous sites in the chicken genome. Given that the chicken sequence itself is estimated to be only about 95% complete [5], it is remarkable that about 90% of the turkey BES mapped to it (and over 90% of these to unique locations). With the rapid reduction in BES sequencing costs, the second approach is attractive in situations where a highly orthologous reference sequence already exists. It risks making false alignments between the test (turkey) and reference (chicken) genomes, but, in general, it more accurately aligns individual BACs within a given contig, is less sensitive to collapsing contigs due to repetitive fingerprints, and avoids false negative overlaps. When additional data (fingerprint maps, overgo hybridization, FISH) are employed to insure consistency, the likelihood of incorrectly aligning genomes (i.e., "chickenizing the turkey genome") appears to be low. In our hands, the second approach decreased the contig number by about an order of magnitude (74 vs. 720). However, this gain in power is somewhat misleading, as in the second approach we performed several rounds of gap-targeted overgo hybridizations to merge contigs, whereas the first approach did not employ iterative gap-filling experiments. It is noteworthy that in our map we were able to place some turkey genome segments that are orthologous to unplaced chicken sequence contigs and to place turkey sequence orthologous to an unplaced chicken linkage group on MGA27 (Additional file 5: Figure S1). Thus, our turkey-chicken comparative map provides information of value towards improving the current chicken sequence assembly.

### Chromosome evolution differentiating the turkey and chicken genomes

Our comparative map predicts 20 to 27 major rearrangements (those involving ~100 kb or more) between the turkey and chicken genomes, mostly inversions (Table 3, Additional files 3 and 5). This result suggests a very high level of stability within Phasianid genomes. The uncertainty in the number of predicted inversions derives in part from the fact that for two chromosomes (MGA12/GGA10 and MGA25/GGA23) the maps are consistent with either two sequential inversions or simultaneous loss/inactivation of an internal centromere and gain/activation of a telomeric centromere. In addition, we were not able to completely resolve a complex alignment pattern of GGA12p to MGA14 that appears to involve at least one large segmental duplication (in turkey) and uncertainty in the respective centromere locations, which may result from one to four inversions. Below, the turkey-chicken chromosome alignment is described on a chromosome-by-chromosome basis.

#### MGA1/GGA1

Despite being by far the longest chromosomes in turkey and chicken, MGA1 and GGA1 are almost completely co-linear. Small differences are observed in the *SEMA3 *gene cluster (9.71-10.05 Mb in WUGSC2.1/galGal3 found at 74.594 Mb in MGA1), and in the *EPHA *gene cluster at around 94 Mb (Additional file 3: Table S3) that may be due to errors in the chicken sequence, segmental duplication differences or possible unequal recombination. There is also a small segment of ribosomal RNA-encoding DNA (rDNA) within the chicken sequence at 104.45-104.85 Mb that is missing in turkey and seems likely to be an assembly error in chicken. Small inversions (at 75.87-75.93 Mb and at 172.82 Mb within WUGSC2.1/galGal3) may also be assembly errors or true rearrangements. Finally, there are two small segments of about 50 and 5 kb at 125.9 Mb and 156.6 Mb, respectively, on MGA1 whose nearest homologues in chicken are on GGA4 (Additional file 5: Figure S1). It seems most likely that these do not represent true translocations but rather were paralogous duplications in the last common ancestor of chicken and turkey, of which chicken inherited one and turkey the other copy or segments that moved due to transposable elements. Both GGA4 segments contain fairly long CR1 retrotransposon sequences.

#### MGA2/GGA3

**Figure 1:**
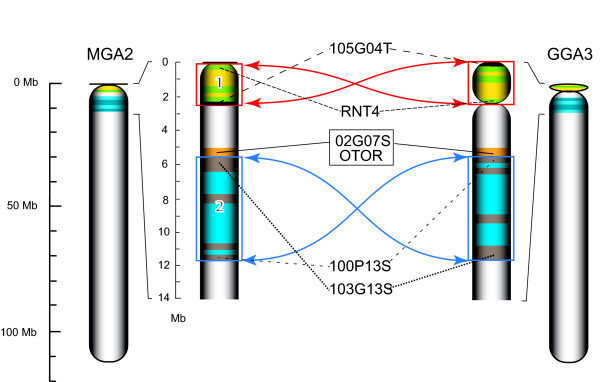
**Turkey chromosome 2 (MGA2) rearrangements**. Chromosome segments are shaded to indicate relative directionality: inversion 1, green and yellow; inversion 2, blue and brown. Arrows (red, inversion 1; blue, inversion 2) connect the inverted segment edges. Constrictions indicate the likely centromere locations. Selected overgo probe marker hybridizations from those supporting the rearrangements are shown. BES mate pairs whose spacing and strandedness support the two proposed rearrangements are listed. Inversion 1 is supported by markers RNT4 and 105G04T as well as 7 paired-end BES matches (Additional file 4: Table S4). Inversion 2 is supported by markers 100P13S and 103G13S, together with 6 paired-end BES spanning the breakpoint. DNA markers 02G07S and OTOR are located at the breakpoint of inversion 2 as landmarks.

**Figure 2:**
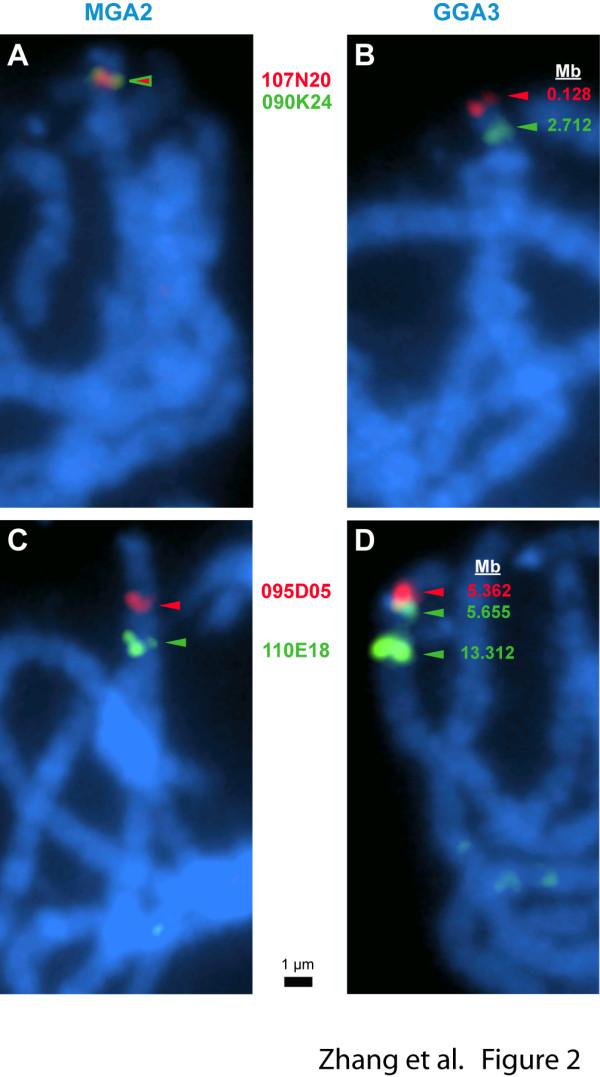
**FISH to pachytene chromosomes of turkey and chicken illustrating the MGA2/GGA3 inversions**. Co-hybridization with CHORI-260 107N20 (red, WUGSC2.1/galGal3 BES coordinates 128288-325088) and 090K24 (green, 2711577-2958331) to turkey pachytene chromosomes gives overlapping signals (A) displayed by color-coded arrowheads, whereas with chicken pachytene chromosomes (B), 107N20 is telomere-adjacent and separated from 090K24 by about 2.5 Mb, as would be expected from the chicken sequence BES alignments. This is consistent with the p-arm inversion on MGA2 relative to GGA3 (Figure 1). Additional FISH with the TM1 centromere probe (unpublished observation) confirms that the MGA2 centromere is telomere-adjacent. Co-hybridization with CHORI-260 095D05 (red, WUGSC2.1/galGal3 BES coordinates 5361781-5538321) and 110E18 (green, 5654670-13311876) to turkey pachytene chromosomes (C) gives distinct, well-separated signals, whereas with chicken pachytene chromosomes (D), the 110E18 signal (green arrowheads) is split, as expected from its inconsistently spaced BES alignment, with one signal adjacent to the 095D05 hybridization (red arrowhead) and the other signal internal on MGA2. The p-arm of MGA2 (A, C) and GGA3 (B, D) is oriented toward the top of each image. These results support inversion of the turkey segment orthologous to the 5.6-11.6 Mb portion of GGA3, as shown in Figure 1. The chicken sequence coordinates of the BACs used as probes are indicated alongside the GGA3 bivalents shown in B and D. Scale bar = 1 μm.

Two inversions differentiate these orthologues. First, the entire GGA3p arm is inverted, and there is also an inversion of the 5.6 to 11.6 Mb segment (Figure 1). The p-arm inversion is supported by 7 BACs whose BES mate pairs span the rearrangement and 17 BACs that hybridize to both *RTN4 *and 105G04T, that flank the breakpoint in turkey, but are separated by 2.45 Mb on GGA3 (Additional file 4: Table S4). The internal inversion is supported by 6 turkey BACs whose BES span the breakpoint and 10 BACs that hybridize to flanking markers in turkey that are separated by 7.5 Mb in chicken. Both rearrangements were confirmed by FISH mapping (Figure 2). The WUGSC2.1/galGal3 sequence assembly had previously located the GGA3 centromere at 11.6 Mb, but Zlotina et al. [38] showed that this site is instead a cluster of repeats. Thus, MGA2 is spanned by a single contig with little or no observable p-arm.

#### MGA3/GGA2q

These two chromosomes differ by an inversion at the p terminus of MGA3 (centromere adjacent on GGA2q, 53.8-56.56 Mb, Additional file 5: Figure S1). In addition, it appears the WUGSC2.1/galGal3 sequence may have misplaced short segments at 54.3 Mb and 54.4 Mb (within the *CNTNAP *gene cluster).

#### MGA4/GGA4q

Other than the two small GGA4 sequence segments found on MGA1 as described above and an apparent small duplication of a segment at 35.16 Mb, these two chromosome arms are co-linear.

#### MGA5/GGA5

**Figure 3:**
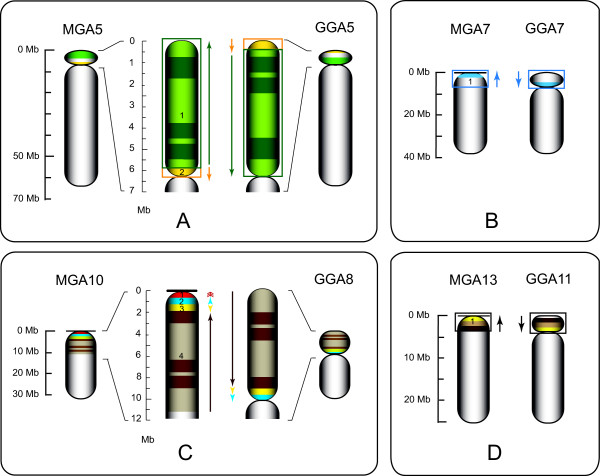
**Turkey chromosome p-arm inversions identified in MGA5, MGA7, MGA10 and MGA13**. Relevant chromosome segments are shaded. Large segments are banded to indicate directionality, also shown by colored arrows flanking enlargements. Centromeres are shown by constrictions. **A**. MGA5. MGA5p is inverted with respect to GGA5p with an additional small inversion of fragment 2 (yellow). Centromere location in MGA5p is tentative (see Results and discussion). **B**. MGA7. GGA7p is inverted with respect to MGA7 (fragment 1, direction indicated by blue arrow) with movement of the centromere to a telomeric site in MGA7. **C**. MGA10. GGA8p is inverted with respect to MGA10 with movement of the centromere to a telomeric site in MGA10. In addition, fragment 3 (yellow) is also inverted (such that its direction is now the same in both species), and we place a turkey genome segment (fragment 1, red) orthologous to a 340 kb fragment from GGA8_random at a telomere-adjacent location on MGA10. **D**. MGA13. GGA11p is inverted with respect to MGA13 with movement of the centromere to at or near the telomere in MGA13.

Based on the genetic map, it appears that the p-arm of MGA5 is inverted relative to GGA5 (Figure 3). Our data do not rule out the possibility that the centromere on MGA5 also became telomeric as part of this inversion; however, the turkey karyotype [4] clearly shows a visible p-arm on MGA5. There is also a small inversion of the p terminal 0.4 Mb of GGA5p (which is now centromere proximal on MGA5).

#### MGA6/GGA2p

Some or all of the p terminal 0.27 Mb of GGA2p appears to map internal to MGA22 (orthologous to GGA20). While this might be due to a translocation, it seems more likely to be an assembly error due to a zinc finger gene family at around 0.3 Mb. Otherwise, MGA6 and GGA2p are completely co-linear. Note that technically, the sequence coordinates of MGA6 should be reversed relative to those of GGA2p since its centromere is now at the distal (high coordinate) end due to the centric fission that generated this chromosome [4].

#### MGA7/GGA7

These orthologues appear to be co-linear except for another p-arm inversion that places the MGA7 centromere near the p terminus (Figure 3). This result is unexpected since MGA7 has a cytogenetically distinguishable p-arm [4]. It is possible that the MGA7 centromere is located at the 10.65 Mb contig gap (Additional file 3: Table S3), although we did not observe multiple repetitive BES in this region as is usually the case adjacent to centromeres. Another possibility is that the MGA7p-arm is mainly repetitive DNA not found in the GGA sequence assembly or that one of the unassembled chicken microchromosomes is fused to the GGA7 sequence to form MGA7p. (Given their respective chromosome numbers and the two known fission events in turkey vs. chicken, there should be at least one fusion of a microchromosome to another chromosome in turkey [4].)

#### MGA8/GGA6

A complex series of rearranged segments are found at the centromeric termini of these two orthologues. Most of these have been confirmed by FISH hybridization (unpublished observations). Although it is impossible to discern the exact order of events without detailed mapping of other Phasianid genomes, it is feasible to explain the various changes by a series of 4 consecutive inversions, each of which had different end points, leading to 8 genome segments whose orientations now differ between the two orthologues (Additional file 5: Figure S1).

#### MGA9/GGA4p

These two orthologues appear to be completely co-linear. As with MGA6, the coordinates for MGA9 should technically be reversed, as the centromere is presumed to remain at the distal (high coordinate) end due to the likely centric fission that generated this chromosome [4].

#### MGA10/GGA8

The p-arm inversion that is the major distinguishing feature of these two orthologues was known from karyotype studies and confirmed by Griffin et al. [39]. As we noted previously [13], the end points of this inversion are consistent with it being due to unequal recombination between the two *amylase *gene paralogues described by Benkel et al. [40]. There is also a ~1.4 Mb segment (Figure 3) within this region that has inverted again such that its direction is now the same in both species. Finally, we have placed orthologous sequence to that found in chr8_random in the chicken sequence assembly at the distal terminus of MGA10, presumably adjacent to the centromere on this telocentric chromosome (Additional file 5: Figure S1).

#### MGA11/GGA9

These two telocentric orthologues differ only by an inversion at the centromeric end, about 3 Mb in length (Additional file 5: Figure S1).

#### MGA12/GGA10

These two orthologues are co-linear, but the MGA12 centromere is now at the distal end, whereas it is about 1.9 Mb internal in GGA10. Several turkey BES mate pairs span the location of the chicken centromere on GGA10 (Additional file 4: Table S4). FISH mapping (unpublished observations) confirms the correct placement of the GGA10 centromere in the sequence assembly but its absence at this site in MGA12. This could be due to two consecutive inversions or to centromere translocation, e.g., replacement of centromere function at the telomere and loss of the interstitial centromere.

#### MGA13/GGA11

The inversion of the GGA11 p-arm leading to MGA13 being telocentric (Figure 3) and its confirmation by FISH have been described previously [41].

#### MGA14/GGA12

As with MGA8/GGA6, there is a complex set of rearrangements at or near the p terminus of this pair. This includes an apparent segmental duplication of a small region containing the *RNF123 *gene that appears in three separate locations in our map. The interstitial centromere in GGA6 has moved closer to the chromosome terminus. FISH resolution was not adequate to determine whether the MGA14 centromere was terminal or between Contig14-1 and 14-2 (Additional file 3: Table S3).

#### MGA15/GGA13

This pair is mostly co-linear except for a small (0.3 Mb) inversion near 8.2 Mb and a possible very small inversion or rearrangement near the centromere. The latter region is problematic in both our map and the chicken sequence due to being the site of the *protocadherin *gene cluster.

#### MGA16/GGA14

**Figure 4:**
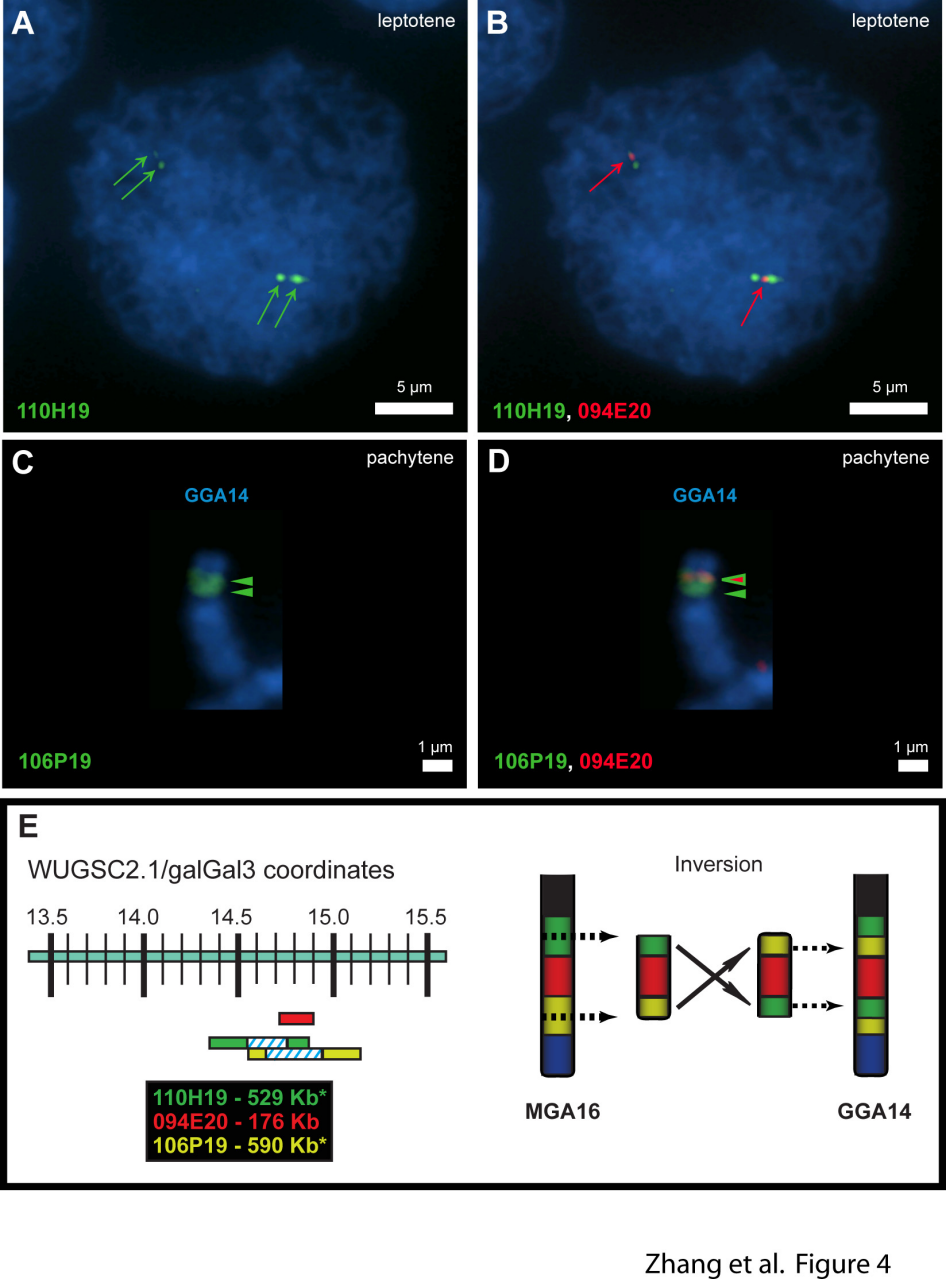
**FISH analysis using turkey BAC probes shows an inversion of 0.7 Mb between MGA16 and GGA14**. CHORI-260 BAC 110H19 exhibits split signals (green arrows) in chicken, (**A**) leptotene stage nucleus and (**C**) pachytene bivalent FISH, as predicted by its widely spread and same strand BES matches to the chicken sequence (WUGSC2.1/galGal3 14315000-14844336). The FISH signal from CHORI-260 BAC 094E20 (red arrows, WUGSC2.1/galGal3 14715031-14890809) aligns consistently with an unbroken sequence mostly internal to the two 110H19 ends and overlapping with one of them on a (**B**) leptotene stage nucleus (same cell as A) and (**D**) pachytene (same bivalent as C). (**E**) Diagram of the inversion between MGA16 and GGA14 based on FISH analysis, overgo hybridization and BES alignments. Chicken sequence coordinates of the BACs are as shown. Asterisks: BES aligned too far apart and to the same DNA strand in the chicken sequence for CHORI-260 110H19 and 106P19. A, B: scale bar = 5 μm; C, D: scale bar = 1 μm.

This pair contains a single internal inversion of about 0.7 Mb around 14.4 Mb that has been confirmed by FISH mapping (Figure 4).

#### MGA17-19/GGA15-17

No rearrangements were detected between any of these three pairs of orthologues. However, both the sequence assembly and our map are incomplete for the very small, rDNA-containing, MGA18 and GGA16. See Reed et al. [37] for a more complete description of MGA18.

#### MGA20/GGA18

As described previously [13], these two chromosomes are distinguished by a large inversion that terminates in oppositely transcribed *NME *gene paralogues, consistent with it being due to unequal recombination.

#### MGA21-26, 28, 29/GGA19-24, 26, 27

To the best of our resolution, these eight orthologous pairs are co-linear. As noted above, we find the telomeric ~0.27 Mb assembled in WUGSC2.1/galGal3 on GGA2p to be located on MGA22 (near the 9.3 Mb coordinate of GGA20). We also were able to position the segment assembled as GGA22_random at ~2.8 Mb on MGA24. Finally, our map shows that the MGA25 centromere cannot be at the orthologous location to that predicted for GGA23 (~1.9 Mb). It remains possible that the MGA25 centromere is close to 3.1 Mb, the break between the two contigs in our map (Additional file 3: Table S3), but we did not observe the pattern of repetitive BES in this region that is typically found near centromeres. Thus, it seems more likely that MGA25 is telocentric (Additional file 5: Figure S1).

#### MGA27/GGA25 and MGA30/GGA28

These two orthologous pairs cannot be accurately aligned, primarily because they are very poorly represented in BAC libraries, as well as in sequence libraries. Gordon et al. [36] showed that the WUGSC2.1/galGal3 chicken sequence assembly was rather inaccurate for GGA28, and we believe the same to be true for GGA25. In general, our map of MGA30 agrees with the Gordon et al. [36] GGA28 sequence, although the map is complicated by an apparent duplication of at least one small segment. We were able to place the turkey orthologous sequence to GGA28_random in our map of MGA30, and we also placed the orthologous turkey sequence to linkage group chrE22C19W28_E50C23 within MGA27. It remains uncertain, but it seems likely that these two unplaced sequences are in similar, if not identical, locations in the chicken genome. The poor coverage of these two chromosome pairs and MGA18/GGA16 also applies to the remaining chicken and turkey microchromosomes and, for this reason, they are not assembled in the chicken sequence nor can they be assigned in our comparative map.

#### MGAZ/GGAZ

**Figure 5:**
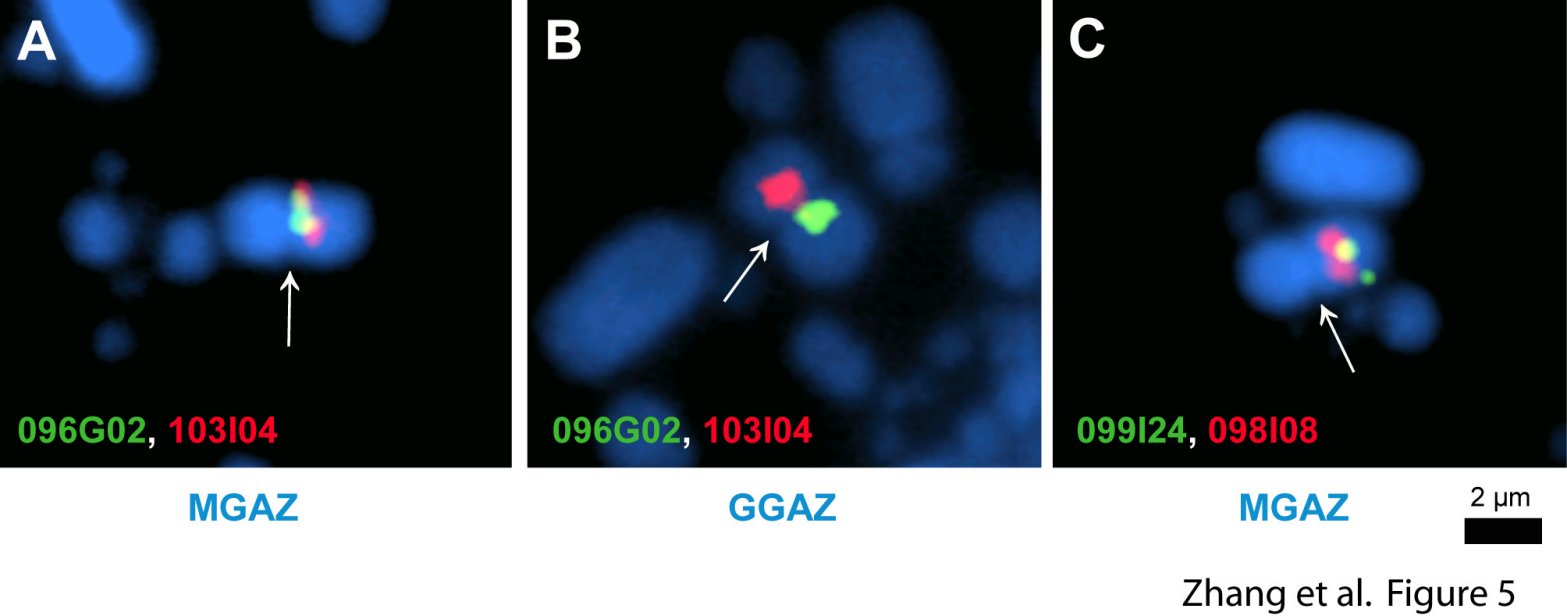
**FISH analysis of MGAZ/GGAZ illustrates a large Z chromosome inversion**. CHORI-260 BACs 096G02 (green, WUGSC2.1/galGal3 coordinates 39290768-39535155) and 103I04 (red, 59681476-59892200) nearly co-localize on a metaphase turkey chromosome with the MGAZ centromere (**A**), but are well-separated on opposite arms on GGAZ (**B**), as would be expected from their chicken sequence coordinates. Shang et al. [42] located the GGAZ centromere at 42.2-42.5 Mb. (**C**) CHORI-260 BAC 099I24 (green, WUGSC2.1/galGal3 coordinates 64731310-64992979) labels the central portion of the second MGAZ arm (the two arms are of nearly identical length), as would be predicted based on the GGAZ sequence of Bellott et al. [35]. However, CHORI-260 098I08 (red, WUGSC2.1/galGal3 coordinates 49538900-49816462) nearly co-localizes with 099I24. These results are consistent with an inversion between the two Z chromosomes spanning at least 49-60 Mb. Overgo hybridization and BES alignment suggest a single ~19 Mb inversion from about 43.9 Mb to 62.8 Mb. Arrows point to the centromere for each chromosome. Scale bar = 2 μm.

The birds used for both the chicken sequence and turkey libraries were female (ZW). Therefore, the sex chromosome maps rely on half the coverage of that for autosomes. Furthermore, Bellott et al. [35] showed that the GGAZq terminus is rich in segmental duplications and poorly assembled in the WUGSC2.1/galGal3 chicken sequence, so this area is difficult to align in our map. The repeats at about 71.5-72.1 Mb in WUGSC2.1/galGal3 and about 81.0 Mb in Bellott et al. [35] are in a location consistent with the block of Z heterochromatin present in the *Phasianidae *but not other land fowl [4]. Also, Shang et al. [42] showed that the centromere is incorrectly located in the WUGSC2.1/galGal3 GGAZ assembly. Our map agrees with their centromere location. The major difference between the two Z chromosomes is a large (~19 Mb) inversion on the second arm (the chicken Z is almost exactly metacentric, complicating definition of a p- and q-arm) extending from about 44 to 63 Mb, using the WUGSC2.1/galGal3 coordinates. This has been confirmed by FISH mapping (Figure 5). There is also a repetitive region near 30.0 Mb that is possibly mis-assembled or contains one or two very short inversions, as well as small segments from chrUn_random and chrZ_random in the sequence, and a segment (ContigZ-7, Additional file 3: Table S3) adjacent to the centromere that may be misplaced in the chicken sequence or may have moved in turkey.

#### MGAW/GGAW

Due to its highly repetitive nature, the GGAW sequence is almost entirely unassembled, and no attempt was made to align it with MGAW.

### Integrating the BAC map with the whole genome shotgun sequence

An earlier version (326 contigs) of our comparative turkey-chicken BAC map provided a critical resource to aid in assembling the turkey genome sequence [13] and aligning it on turkey autosomes (our Z map was too preliminary to use at that time). This is particularly important for the rapidly expanding set of genomes, like that of the turkey, that are shotgun sequenced solely by "next-generation" methodologies that tend to give shorter sequence contig and scaffold lengths. On the other hand, high quality BAC physical/comparative maps provide an even more critical tool for those genome sequences generated by BAC sequencing of minimal tile paths with either Sanger-based or pooled next generation sequencing technologies [43,44]. While we did not use the turkey sequence assembly to improve the turkey BAC contig map in order to avoid conclusions based on circular reasoning, there are a small number of the remaining contig gaps that could now be merged based on turkey sequence scaffold or contig data (Additional file 3: Table S3). Such gaps most likely derive from short regions where there was little or no BAC coverage, despite the use of the two large-insert libraries. Although genetic maps [25,26] also provide long range data that were used to align the turkey sequence scaffolds along chromosomes, their resolution is limited by marker density and/or linkage disequilibrium, and, in our experience, they are less accurate than a comparative map based on dense alignment of BACs using BES and/or overgo hybridization, along with BAC fingerprint contigs.

## Conclusions

The turkey-chicken BAC map leads to several general conclusions. First, it confirms observations that avian genomes (at least those within the *Phasianidae*) show a very high level of stability [45,46]. Despite as much as 40 million years of separate evolution since their last common ancestor, there may be as few as 20 and likely no more than 27 substantive (> 100 kb) rearrangements separating the two genomes (Table 3), not including those microchromosomes and the W chromosome that have yet to be accurately assembled in either species. Second, those rearrangements that are observed appear to be almost totally due to intra-chromosomal inversions. Although we observed a few instances of possible translocation events, based on their size, it appears that these are much more likely to be due to transposable element action, chicken sequence mis-assembly or duplicated sequences in the last common ancestral genome that were differentially inherited by chicken and turkey. We find no evidence in our map to support the two inter-chromosomal rearrangements proposed by Aslam et al. [26] based on turkey single nucleotide polymorphism (SNP) mapping nor can we confirm very many of their proposed 57 intra-chromosomal rearrangements, other than the inversions between MGA10/GGA8 and MGA20/GGA18 that we described previously [13]. Although the reasons for this discrepancy are uncertain, examination of the rearranged SNP loci suggests that several occur in duplicated sequences or within, or adjacent to, transposable elements. This would be consistent with movement of small sequence segments via transposition or differential inheritance of paralogous duplications that were present in the last common ancestral genome. Although the reasons for the predominance of inversion events are uncertain, it is noteworthy that at least two appear to arise from unequal recombination between duplicated genes arranged in inverted order on the same chromosome (MGA10, *amylase *and MGA20, *NME *genes). It therefore seems possible that some of the others are due to unequal recombination between CR1 elements or other repetitive sequences.

These results strongly support the surprising conclusion that there is a clear trend in turkey towards telocentric chromosomes, i.e., centromeres directly adjacent to the telomere. Figures 1, 2 and 3, and Additional file 5: Figure S1 show that interstitial centromeres of chicken chromosomes are located proximal to telomeres in their turkey orthologues for MGA2, MGA7, MGA10, MGA12, and MGA13 and possibly in MGA14 and MGA25. In addition, the two centric fission events involving GGA2 and GGA4 [4,46] that produce MGA3, MGA4, MGA6 and MGA9 all result in telocentric turkey chromosomes. Indeed, the only clearly metacentric chromosomes in turkey are MGA1 and the sex chromosomes, MGAZ and MGAW. MGA5 may have a small p-arm, and MGA7 clearly has a p-arm visible in the karyotype [4]. However, we were unable to map any sequence orthologous to the chicken genome to MGA7p. The reason, if any, for this trend away from interstitial centromeres in turkey is unclear. Based solely on karyotype data [4], it would appear that a predominance of telocentric chromosomes is mostly a derived trait within turkey and closely-related pheasants, but cytogenetics alone cannot distinguish several of the rearrangements we have documented, so further comparative mapping would be required to clearly delineate this trend in Phasianid evolution.

The comparative BAC contig map, along with other genomic resources previously developed in the species, provides the foundation necessary for many areas of advanced genomics research in turkey, chicken and other *Galliformes *species. For the turkey, as for other agricultural animal and crop species, a primary area of interest is trait (often QTL) analysis based on linkage maps, increasingly derived using high density SNP arrays. The BAC-based physical map provides an essential resource for additional molecular analysis of trait loci and for positional cloning.

## Methods

### Source BAC libraries

A new turkey BAC library, TKNMI, was constructed for this study with DNA from the same female turkey employed for the CHORI-260 BAC library and for genome sequencing [13] using methods described previously [47,48]. TKNMI is based on insertion of *Mbo*I partial digest fragments into the pECBAC1 vector and contains 46,080 clones. Analysis of 100 random clones showed an average insert size of 160 kb (Additional file 6: Figure S2), and TKNMI thus provides 6.7-fold coverage of the turkey haploid genome. Fewer than 5% of TKNMI clones contain no inserts. The TKNMI library was used in combination with the pre-existing CHORI-260 library (*Eco*RI inserts into pTARBAC2.1, average insert size of ~190 kb [13]) for map development. A total of 85,208 turkey BAC clones were randomly selected from the two BAC libraries for map construction, covering the haploid turkey genome about 13.7-fold. The CHORI-260 BAC library is publicly available through the Children's Hospital of Oakland Research Institute BACPAC Resources Center [49]. TKNMI is publicly available through the Laboratory for Plant Genomics and *GENE*finder Genomic Resources at Texas A&M University, College Station, Texas [50].

### BAC DNA preparation and fingerprinting

Our previous studies demonstrated that BAC fingerprints generated with different restriction enzyme combinations result in different quality physical maps [33]. Therefore, we first tested twenty-four 3-, 4- and 5-enzyme combinations of *Bam*HI, *Eco*RI, *Hin*dIII, *Xba*I, *Xho*I, and *Hae*III on 96 BACs randomly selected from the TKNMI library. Only the ends produced by *Bam*HI, *Eco*RI, *Hin*dIII, *Xba*I or *Xho*I digestion were labeled (using NED-ddATP or HEX-ddATP, see below). *Hae*III digests the labeled fragments to sizes that allow separation on a capillary sequencer. Criteria employed were that there is no partial digestion, no star activity, an average of 35-70 bands per clone and a relatively even size distribution of the bands in a window ranging from 35 - 500 base pair (bp). The enzyme combination of *Bam*HI/*Eco*RI/*Hae*III was selected for generation of BAC fingerprints for the turkey BAC libraries. Turkey BAC clones arrayed in 384-well microtiter dishes were inoculated into 96-deep well plates containing 1.0 ml TB (Terrific Broth, [51]) medium with appropriate antibiotics using a 96-pin replicator (BOEKEL, Feasterville, PA, USA). The 96-deep well plates were covered with air-permeable seals (Excel Scientific, Wrightwood, CA, USA) and incubated in an orbital shaker at 300 rpm, 37°C for 18-22 h. Overnight cultures were centrifuged at 3,000 g for 10 min in a Beckman bench-top centrifuge to harvest cells. BAC DNA was isolated using a modified alkaline lysis method [51], dissolved in 15 μl TE (10 mM Tris-HCl, pH 8.0, 1 mM EDTA, pH 8.0) with 8 U/ml RNaseA, 320 U/ml RNase T1 (Applied Biosystems, Foster City, CA, USA) and stored at -20°C before use. DNA was digested and end-labeled in a reaction containing 50 mM NaCl, 10 mM Tris-HCl, 10 mM MgCl2, 1.0 mM dithiothreitol, pH 8.0, 1.0 mM dNTP, 1.0 μg/μl BSA, 1 U each of *Bam*HI, *Eco*RI, and *Hae*III (New England Biolabs, Ipswich, MA, USA), 0.3 U *Taq *FS and 6.0 μM HEX-ddATP or NED-ddATP. The reaction was incubated at 37°C for 2 h, followed by further incubation at 65°C for 45 min. DNAs labeled with different fluorescent dyes (HEX-ddATP or NED-ddATP) were combined, pelleted, washed, dried and dissolved in a mixture of 9.8 μl of Hi-Di formamide and 0.2 μl of the internal GeneScan-500 Rox size standard (Applied Biosystems). DNA was denatured at 95°C for 3 min, cooled on ice and then subjected to analysis on an ABI 3100 Genetic Analyzer (Applied Biosystems) using the default GeneScan module. A total of 85,208 turkey BAC clones were randomly selected from the two BAC libraries and fingerprinted for physical map construction. BAC fingerprint fragment sizes were determined using the ABI Data Collection program (Applied Biosystems). ABI 3100 Genetic Analyzer data were processed using the software package ABI-ExportTabularData [52] and SeqDisplayer (unpublished). Data were transformed using an automatic algorithm by SeqDisplyer into "bands" files. Several quality checks were applied to the fingerprints, with sample-empty wells being removed, fingerprints with fewer than 15 band peaks removed, background peaks identified and removed, off-scale bands with peak heights greater than 6,000 removed, and vector band peaks removed. Only the bands falling between 35 and 500 bp were used.

### BAC contig physical map assembly

Fingerprints of 74,013 (87%) clones were validated and used for physical map assembly, corresponding to approximately 11.9-fold coverage of the turkey genome. Clones had an average of 53.9 restriction fragment bands in the window of 35 - 500 bases, with a range from 15 to 320 bands per clone. According to our previous studies [33,34], 11.9-fold genome coverage should suffice for assembly of a high-quality genome-wide physical map of the turkey genome. FingerPrinted Contig (FPC) version 9.3 [27] was used to assemble the turkey contig map from the BAC fingerprints. Two parameters, tolerance and cutoff, are crucial to the quality of contig assembly. Tolerance, the window size in which two restriction fragments are considered as equivalent, was set initially by determining the average 95% confidence interval for the mean size deviation for each of four pECBAC1 vector fragments. In addition, tolerances of 4 - 10 were tested using the entire fingerprint dataset to determine the parameters suitable for contig assembly. On the basis of these results, a tolerance of 7 was finally selected. Cutoff values (probability threshold that fingerprint bands match by coincidence) of 1e-20 - 1e-02 were tested using the entire fingerprint dataset, and the resultant numbers of contigs, singletons, and questionable-clones (Q-clones) were analyzed. At higher stringencies (1e-20 to 1e-10), chimeric contigs were split and Q-clones were reduced, but the number of singletons increased drastically (Additional file 7: Figure S3). At lower stringencies (1e-05 - 1e-02), a smaller total number of contigs and larger contigs were obtained, but a larger number of clones fell into the Q-clone category. The relationship among the three factors is shown in Figure S3 (Additional file 7), from which it is apparent that a cutoff value of approximately 1e-08 to 1e-06 resulted in reasonably low numbers for all three outputs, suggesting a high quality contig assembly. On the basis of these results, a tolerance of 7 and cutoff of 1e-08 were ultimately selected for initial contig assembly. The initial build of the turkey physical map resulted in the generation of 8,870 automatic contigs, prior to incorporation of any BES or DNA marker results.

### BAC end sequencing

BAC DNA was isolated and purified as described above. ABI (Applied Biosystems) BigDye Terminator v3.1 Cycle Sequencing Kit reactions contained: 6 μl BAC DNA (100 - 150 ng/μl), 1 μl standard T7 or SP6 sequencing primer (4 pmol), 1 μl BigDye cycle sequencing-ready reaction mix, and 2 μl of ABI 5 × sequencing buffer. Thermal cycling (ABI GeneAmp PCR System 9700, Applied Biosystems) was performed at 96°C for 4 min, followed by 99 cycles of 96°C for 30 s, 56°C for 10 s, 60°C for 4 min; and then 60°C for 7 min, and 4 °C for storage. Reaction products were purified by precipitation in 4 μl of 1.5 mM sodium acetate in 70% ethanol, followed by washing in 200 μl of 70% ethanol. The samples were then loaded on an ABI 3100 Genetic Analyzer. BAC-end sequences were trimmed with Phred software [53,54] using Q ≥ 20 as a cutoff. Repeats were masked by RepeatMasker software [55]. A total of 43,224 BAC ends were sequenced from both CHORI-260 (21,738) and TKNMI (21,486). After vector trimming and quality assessment, 36,941 BES were deposited in Genbank [Genbank: ER942218-ER962259 and FI503157-FI520055] (Additional file 8: Table S5).

### Overgo hybridization probe design and library screening

High-density turkey BAC DNA filters were prepared from the CHORI-260 (73,728 arrayed BACs) and TKNMI (36,864 arrayed BACs) libraries as described previously [56]. The libraries were screened with approximately 4,000 overgo probes by pooled overgo hybridization [57]; CHORI-260 was screened with all the probes, but TKNMI only with a subset of the probes. The overgo probes were designed from turkey BES, microsatellite markers and genes, chicken genes and other regions of the chicken genome demonstrating high evolutionary conservation, and a small number of zebra finch EST sequences as described previously [57]. All overgo hybridization probes were tested in advance using BLAT for a unique alignment with WUGSC2.1/galGal3 (Additional file 9: Table S6). In a few cases, overgo probes matching two closely linked duplicated chicken sequences were employed. Overgo labeling and hybridization were performed as described previously using a redundant 4-dimensional pooling strategy with 216 probes per screen [57-59]. Overgo hybridization resulted in 41,423 BAC assignments to 3,499 successful overgo probes (Additional file 9: Table S6). In early stages of this work, overgo locations were sampled broadly across the genome, whereas later screens were specifically designed to resolve possible rearrangement locations and gaps in the comparative map.

### Map integration

Comparative mapping of turkey BES to the chicken May 2006 (WUGSC2.1/galGal3) genome sequence assemblies [5] was done using NCBI-BLAST [60], requiring matches of 90% or more for at least 50 bp. An expectation value of 1e-05 was used as the significance threshold for comparison of turkey BES with the chicken genome sequence assembly. Alternatively, BES were aligned to WUGSC2.1/galGal3 using BLAT [61] with initial parameters of minScore = 200 and minIdentity = 70. Respectively, 91% and 87% of BES from CHORI-260 and TKNMI mapped to the chicken genome. Of those matches, 91 - 92% mapped to a unique location. Of those BACs for which both ends mapped to unique locations, 96 - 97% mapped "consistently", i.e., BES mapped to sites 10-400 kb apart and on opposite strands in the chicken genome assembly. BES that mapped to chicken chrUn_random were treated as having failed to match. All BACs for which both BES mapped uniquely but to inconsistent locations and BACs for which one or both BES mapped to multiple locations were examined manually. In many cases, a single consistent map location could be identified among the multiple BLAT BES hits that allowed consistent alignment of both turkey BES or resolved a situation identified as inconsistent by the batch alignment. Most of the remaining inconsistent BES paired matches identified sites of rearrangements between the turkey and chicken genomes as confirmed by multiple BAC alignments, overgo hybridization and/or FISH analysis (Additional file 4: Table S4).

### Cytogenetic analysis

Turkey and chicken chromosome harvests were prepared from turkey embryo fibroblast and chicken embryo fibroblast cultures for mitotic metaphase cells and from adult male gonads for meiotic pachytene stage cells; the latter was in order to have extended chromosomes for improved resolution of BAC probe order. The procedures for chromosome harvest, slide preparation, probe labeling, hybridization, and image capture were as described previously [37,62-65]. BAC clones spanning or near putative chromosome rearrangements as predicted from BES and/or overgo hybridization analysis were utilized in multi-color FISH experiments. In each experiment, two to four probes were hybridized, in some cases to both chicken and turkey preparations (in the same experiment). The process was iterative in investigating rearrangements predicted from BES alignments, in that confirmed BACs were then partnered with new test-BACs to resolve questions of order. Some test BACs were found to be unsuitable due to widespread hybridization to multiple locations, presumably due to containing excessive levels of highly repetitive sequences (often near likely centromeres or telomeres).

## Abbreviations

BAC: bacterial artificial chromosome; BES: BAC end sequence(s); bp: base pairs; FISH: fluorescence *in situ *hybridization; FPC: FingerPrinted Contigs; kb: kilobase pairs; Mb: megabase pairs; QTL: quantitative trait locus; Q-clone: questionable clones in BAC contig alignments; rDNA: ribosomal RNA-encoding DNA; SNP: single nucleotide polymorphism

## Authors' contributions

JBD, HBZ and MED designed and coordinated the overall project. YZ, HBZ, JBD, MED and THO prepared the manuscript. YZ, XZ, JJD, CFS, MZ, JJH and MKL prepared the BAC library, performed BAC fingerprinting and generated BAC end sequences. YZ, HBZ and JBD prepared BAC physical and comparative maps. WSP and JBD performed overgo hybridizations. THO and MED performed cytogenetic analyses. All authors read and approved the final manuscript.

## Supplementary Material

Additional file 1 **Table S1**. Turkey BAC physical map contig summary. Turkey physical map contigs are listed with coverage statistics and corresponding chicken chromosome alignment. Click here for file

Additional file 2 **Table S2**. Turkey BACs that aligned to the chicken sequence. Turkey BACs are either from CHORI-260 (prefix = CH260-) or TKNMI (prefix = 78TKNMI-) libraries. Turkey BACs sorted by BAC library and well number are listed with the method of alignment and their most likely ortholgous alignment with the WUGSC2.1/galGal3 sequence assembly by chromosome, start coordinate and range. Turkey BACs were aligned either by overgo hybridization (magenta) or by BES alignment. For the latter, if both BES aligned consistently and uniquely, the row is green. If only one BES was available or could be aligned, the row is blue and the span of the BAC was arbitrarily estimated at 200 kb for CHORI-260 or 150 kb for TKNMI BACs. If two BES were available but one had repetitive matches, the row is tan. If a likely repetitive match was found manually that was consistent with the other unique BES match, then that was chosen as the second BES coordinate; otherwise size was arbitrarily estimated as above. If both BES had unique but inconsistent matches, the row is yellow. In some cases BACs were placed by both hybridization and BES alignment, as shown in two separate rows. Click here for file

Additional file 3 **Table S3**. Turkey-chicken comparative map contigs and coordinates. Turkey BAC contigs are listed in sequence along turkey chromosomes. Contigs are divided into subcontigs (e.g., 1-2.1, 1-2.2, etc.) due to internal rearrangements or duplications with respect to the chicken genome that have been merged by independent overgo hybridization and/or BAC fingerprint contig data (Additional file 4: Table S4). Start and end coordinates of the orthologous WUGSC2.1/galGal3 chicken sequence are given for all subcontigs (columns D and E). Total lengths are listed only for full contigs (column F). Additional notes clarifying subcontig orientation and arrangement or explaining gaps are provided (columns G and H). As indicated, some gaps between adjacent contigs are spanned by turkey shotgun sequence scaffolds [13], but we have not merged contigs on that basis herein. Click here for file

Additional file 4 **Table S4**. Comparative map alignments of turkey BACs to the chicken genome. Turkey BACs are arranged according to their orthologous chicken sequence coordinates (WUGSC2.1/galGal3). See Table S2 legend (Additional file 2: Table S2) for explanation of BAC names and row shading. Contig gaps or rearrangement breakpoints are indicated in unshaded rows, as noted. Column B lists the corresponding contig number for selected BACs in the final (Oct. 2010) BAC FPC physical map assembly (N/A = either no fingerprint available or singleton BAC). Column C lists contig numbers for CHORI-260 BACs only in an earlier (March 2008) FPC assembly (nf = no fingerprint available; si = singleton BAC). Column D lists the computer-estimated BES alignment outcome (sometimes corrected later by manual annotation) or the respective overgo hybridization probe used (magenta rows). Columns E and F list the chicken chromosome and start coordinate, respectively, of the most likely orthologous location (sometimes as corrected by manual annotation); whereas columns G and H list the initial chicken chromosome and range of alignment coordinates, respectively. For BACs identified by hybridization (magenta) only the estimated orthologous coordinates of the probe (~ 40 bp) are shown; the BAC extends in both directions from this site for unknown distances. Columns I provides information on other probes that hybridize to the BAC, other miscellaneous information or, in relevant cases, the corresponding alignments in the Gordon et al. [36] sequence of GGA28 or the Bellott et al. [35] sequence of GGAZ. Click here for file

Additional file 5 **Figure S1**. Summary diagram of the turkey-chicken comparative map. Turkey chromosome segments are depicted by arbitrarily colored arrows (as per Additional file 3: Table S3). Arrow direction corresponds to ortholgous alignment to the chicken genome (WUGSC2.1/galGal3) from low to high coordinate. Segments larger than 1 Mb (A), 0.5 Mb (B) or 0.1 Mb (C) are to scale as shown; smaller segments are not to scale. Centromeres, gray-filled circles, are to scale using the arbitrary sizes chosen in WUGSC2.1/galGal3 (1.5 Mb for GGA1-10 and GGAZ; otherwise 0.5 Mb). Regions of one or more local rearrangement are boxed. (A) MGA1-7, MGA9 and MGAZ. Gray arrows indicate small segments on GGA4 found on MGA1 likely due to transposon movement or GGA assembly errors. The GGA3 and GGAZ centromeres are placed according to [38] and [42], respectively. Asterisks indicate: green, a small fragment of rDNA sequence at 104.45 Mb on GGA1 not in turkey; blue, turkey orthology to the telomeric 0.3 Mb of GGA2p in WUGSC2.1/galGal3 is found at 9.3Mb on MGA22; magenta, a few very small possible inversions and a segment of GGA chrZ_random and of chrUn_random near 30.0Mb on MGAZ; and red, a very small segment at 42.47 Mb of uncertain location and orientation. (B) MGA8 and MGA10-22. (C) MGA23-30. Possible rearrangements between MGA27/GGA25 and MGA30/GGA28 are uncertain due to incomplete chicken sequence assemblies. Click here for file

Additional file 6 **Figure S2**. Analysis of TKNMI turkey BAC library insert sizes. TKNMI BAC DNAs were digested with *Not*I (New England BioLabs, USA) and subjected to CHEF DRIII (Bio-Rad, USA) electrophoresis. M indicates marker lanes containing a lambda phage DNA ladder with sizes as indicated at right. The 7.5 kb band is the pECBAC1 vector DNA found in all lanes. Click here for file

Additional file 7 **Figure S3**. Determination of optimal cutoff values. A series of cutoff values ranging from 1e-2 to 1e-30 with a tolerance of 7 was tested for automatic contig assembly. Filled circles indicate number of contigs, open circles indicate number of questionable clones (Q-clones) and filled triangles indicate singleton number. A cutoff value of 1e-08 was used in ultimate physical map assembly based on all three factors. Click here for file

Additional file 8 **Table S5**. Turkey BES Genbank accession numbers. CHORI-260 BES accession numbers begin with the prefix CH260-, and TKNMI BES accession numbers begin with the prefix 78TKNMI-. Click here for file

Additional file 9 **Table S6**. Turkey BAC-overgo hybridization results. Column B lists overgo probes by the BES or gene or marker name from which they were designed. Column C lists Genbank accession numbers of sequences used for probe design. Columns D, E, and F list coordinates of BLAT alignment of overgo sequence to the WUGSC2.1/galGal3 chicken sequence by chromosome, start coordinate, and range, respectively. Column G lists marker sequence type (BES, turkey EST, chicken genome, etc.). Column H lists hybridizing BACs, with those from TKNMI beginning with "T"; all others are CHORI-260 BACs. The quality of the assignment is estimated at three confidence levels, P = probable, T = tentative, and W = weak. Approximately, P indicates a clear signal on 4 of 4 appropriate overgo pools, T generally indicates either one (of four) fainter or smeared signals and W usually indicates only 3 of 4 dimensions being positive, all hybridizations being faint or some other concern. Unshaded rows indicate overgos made using turkey sequence sources, those shaded in yellow indicate overgos designed from chicken sequences, and those shaded blue indicate overgos designed from zebra finch sequences. Click here for file
