# Supplementary figures and images for: A comparative physical map reveals the pattern of chromosomal evolution between the turkey (Meleagris gallopavo) and chicken (Gallus gallus) genomes
Source: BMC Genomics. 2011 Sep 9;12:447. doi: 10.1186/1471-2164-12-447 (PMC3189400; doi:10.1186/1471-2164-12-447)

**A**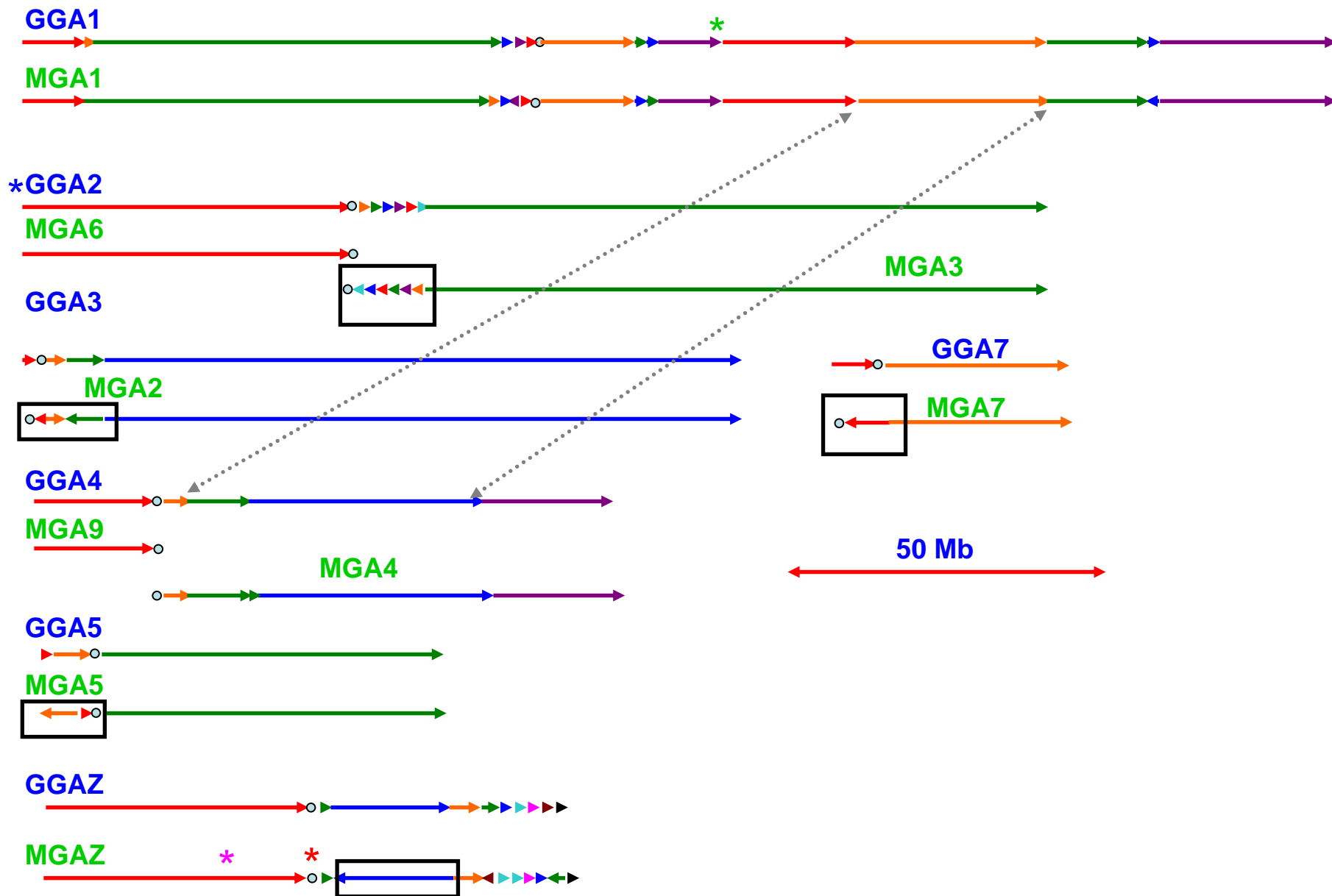

**B**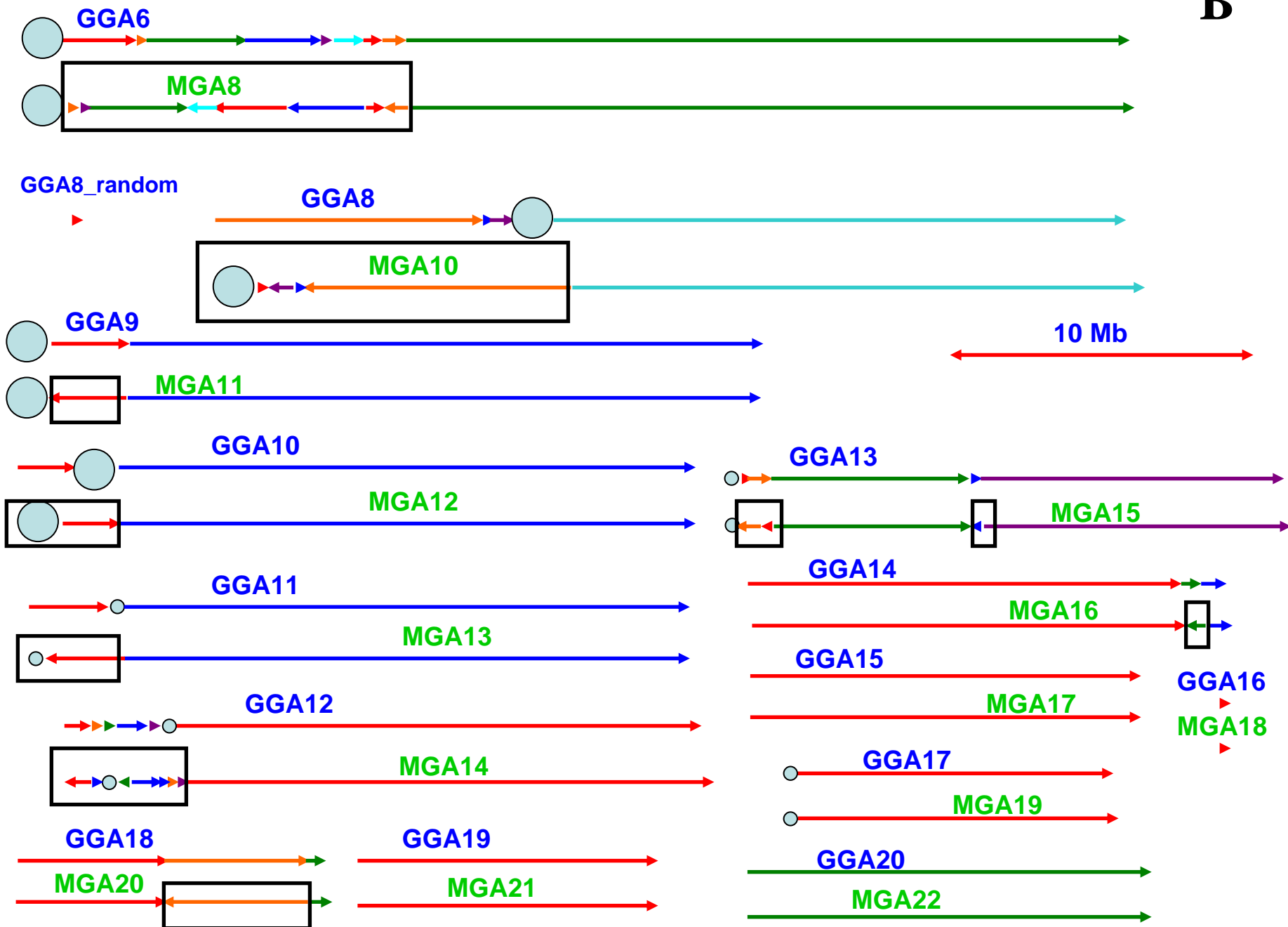

**C**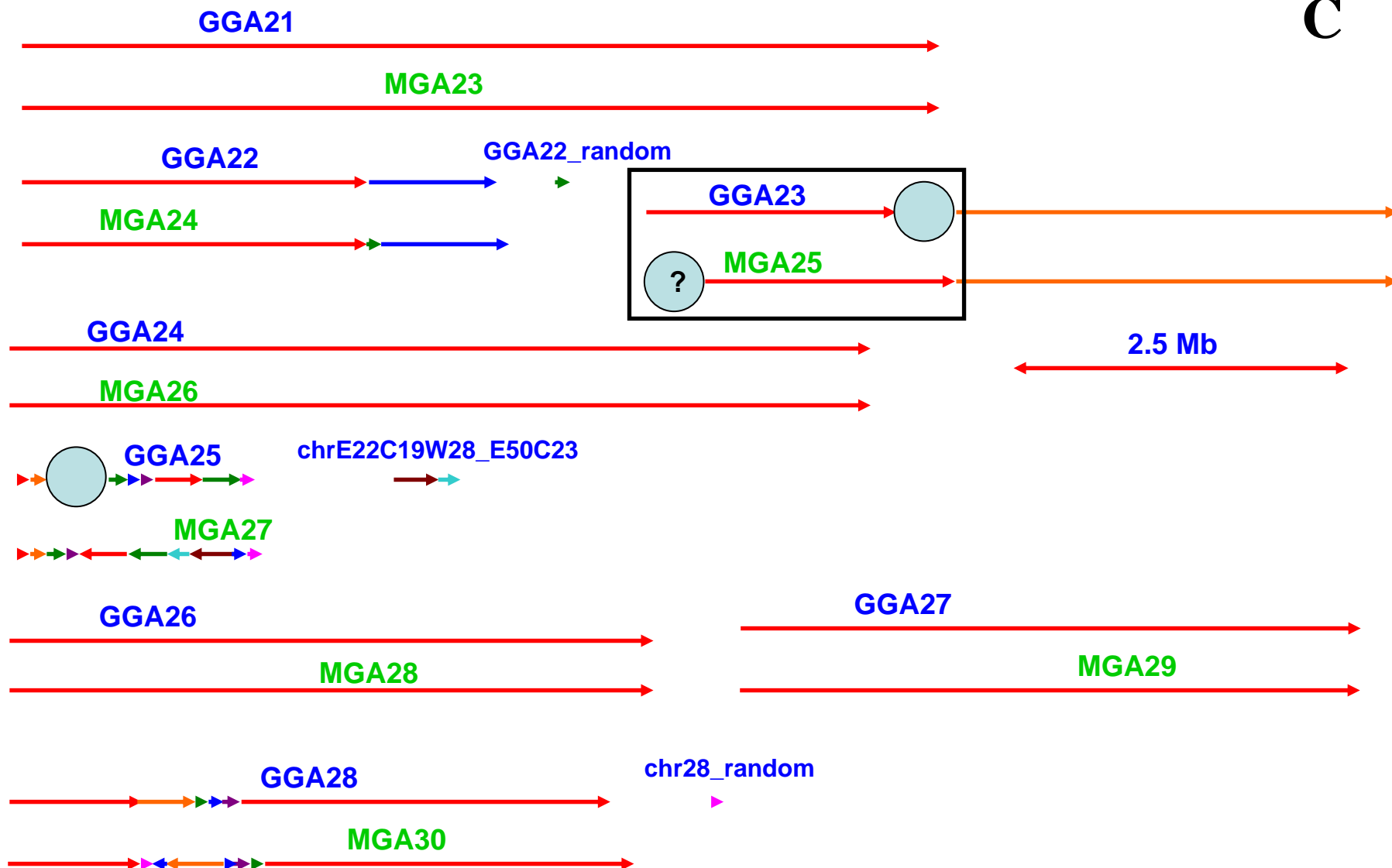

Supplement: Additional file 5 — Figure S1. Summary diagram of the turkey-chicken comparative map. Turkey chromosome segments are depicted by arbitrarily colored arrows (as per Additional file 3: Table S3). Arrow direction corresponds to ortholgous alignment to the chicken genome (WUGSC2.1/galGal3) from low to high coordinate. Segments larger than 1 Mb (A), 0.5 Mb (B) or 0.1 Mb (C) are to scale as shown; smaller segments are not to scale. Centromeres, gray-filled circles, are to scale using the arbitrary sizes chosen in WUGSC2.1/galGal3 (1.5 Mb for GGA1-10 and GGAZ; otherwise 0.5 Mb). Regions of one or more local rearrangement are boxed. (A) MGA1-7, MGA9 and MGAZ. Gray arrows indicate small segments on GGA4 found on MGA1 likely due to transposon movement or GGA assembly errors. The GGA3 and GGAZ centromeres are placed according to [38] and [42], respectively. Asterisks indicate: green, a small fragment of rDNA sequence at 104.45 Mb on GGA1 not in turkey; blue, turkey orthology to the telomeric 0.3 Mb of GGA2p in WUGSC2.1/galGal3 is found at 9.3Mb on MGA22; magenta, a few very small possible inversions and a segment of GGA chrZ_random and of chrUn_random near 30.0Mb on MGAZ; and red, a very small segment at 42.47 Mb of uncertain location and orientation. (B) MGA8 and MGA10-22. (C) MGA23-30. Possible rearrangements between MGA27/GGA25 and MGA30/GGA28 are uncertain due to incomplete chicken sequence assemblies. [file 1471-2164-12-447-S5.PDF]

M

M

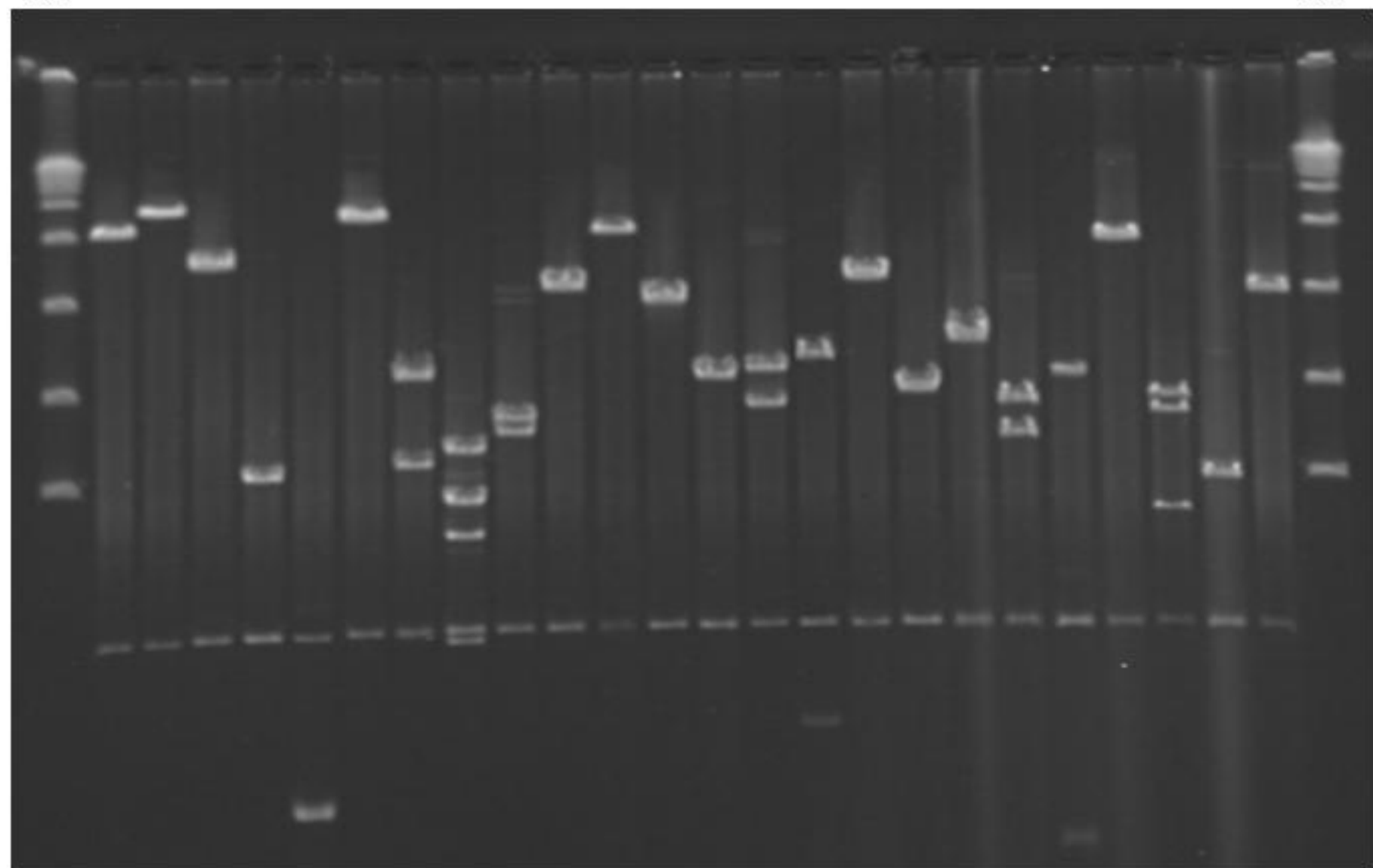

kb

- 197

- 145.5

- 97

- 48.5

- 7.5

Supplement: Additional file 6 — Figure S2. Analysis of TKNMI turkey BAC library insert sizes. TKNMI BAC DNAs were digested with NotI (New England BioLabs, USA) and subjected to CHEF DRIII (Bio-Rad, USA) electrophoresis. M indicates marker lanes containing a lambda phage DNA ladder with sizes as indicated at right. The 7.5 kb band is the pECBAC1 vector DNA found in all lanes. [file 1471-2164-12-447-S6.PDF]

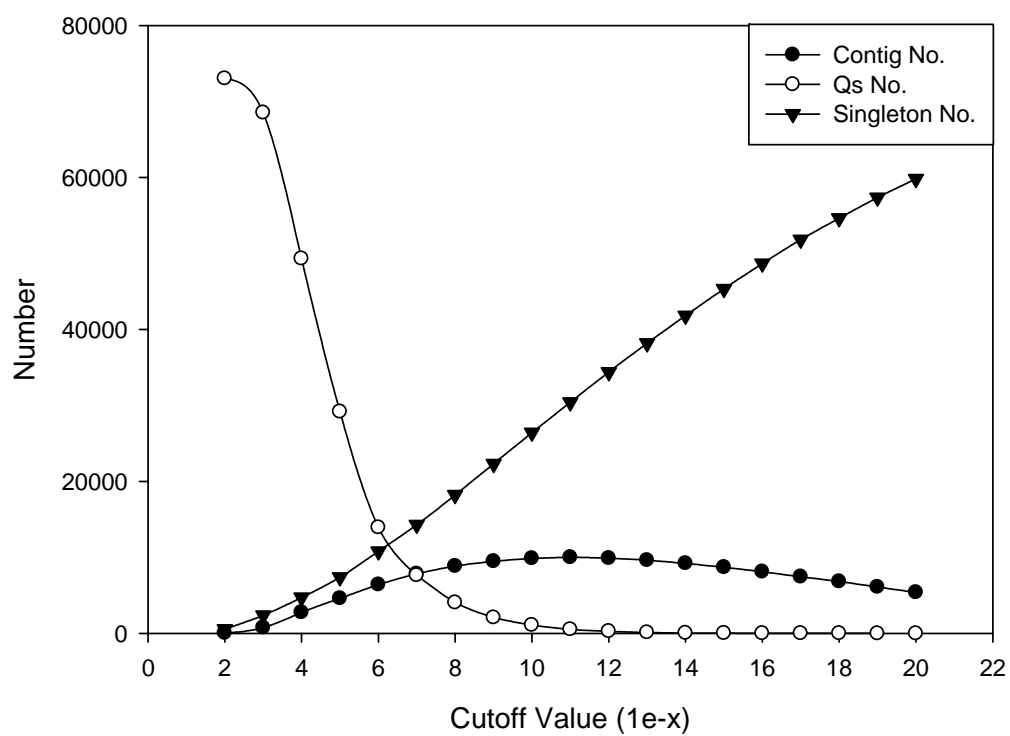

Supplement: Additional file 7 — Figure S3. Determination of optimal cutoff values. A series of cutoff values ranging from 1e-2 to 1e-30 with a tolerance of 7 was tested for automatic contig assembly. Filled circles indicate number of contigs, open circles indicate number of questionable clones (Q-clones) and filled triangles indicate singleton number. A cutoff value of 1e-08 was used in ultimate physical map assembly based on all three factors. [file 1471-2164-12-447-S7.PDF]
